# Why making smoking cessation a priority for rare interstitial lung disease smokers?

**DOI:** 10.18332/tpc/190591

**Published:** 2024-06-28

**Authors:** Cristina Vicol, Raluca Ioana Arcana, Antigona Carmen Trofor, Oana Melinte, Andrei Tudor Cernomaz

**Affiliations:** 1 University of Medicine and Pharmacy “Grigore T. Popa”, Iaşi, Romania; 2 Clinical Hospital of Pulmonary Diseases, Iasi, Romania; 3 Regional Institute of Oncology, Iaşi, Romania

**Keywords:** Interstitial lung disease, smoking-associated diseases, tobacco cessation

## Abstract

This review aims to discuss the complex relationship between smoking and interstitial lung diseases (ILDs), emphasizing the significant morbidity and mortality associated with these conditions. While the etiology of ILDs remains multifactorial, cigarette smoking emerges as a prominent modifiable risk factor implicated in their pathogenesis and progression. This narrative review will provide insight into smoking-associated interstitial lung diseases and personalised approaches to smoking cessation. Epidemiological studies consistently link smoking to ILDs such as idiopathic pulmonary fibrosis (IPF), respiratory bronchiolitis-associated ILD (RB-ILD), and desquamative interstitial pneumonia (DIP), highlighting the urgent need for comprehensive tobacco cessation strategies. Despite the established benefits of smoking cessation, adherence to cessation programs remains challenging due to nicotine addiction, psychological factors, and social influences. The modest success rates of smoking cessation in ILD patients, emphasises the importance of tailored interventions and ongoing support is needed to overcome barriers and to improve outcomes of quitting smoking in this category of vulnerable patients.

## INTRODUCTION

Interstitial lung diseases (ILDs) represent a diverse array of chronic pulmonary conditions characterized by inflammation and fibrosis within the lung parenchyma, resulting in progressive respiratory decline and impaired gas exchange. Despite advances in diagnostics and treatments, ILDs remain a challenge for clinicians and researchers worldwide, associated with significant morbidity and mortality rates across various subtypes^1^.

While the precise etiology of ILDs remains elusive, there is increasing recognition of their multifactorial nature, involving a combination of genetic predisposition, environmental exposures, and immune dysregulation. Notably, cigarette smoking has emerged as a prominent modifiable risk factor implicated in the pathogenesis and progression of certain ILDs. The relationship between smoking and ILDs has gained considerable attention, supported by epidemiological observations, experimental studies, and clinical evidence suggesting tobacco exposure’s role in pulmonary fibrosis, bronchiolitis, and other interstitial lung disorders^2^.

Epidemiological research has consistently demonstrated a strong association between smoking and ILDs, particularly in idiopathic pulmonary fibrosis (IPF), where smokers exhibit a significantly increased risk of disease development and progression compared to non-smokers. Additionally, smoking-related ILDs such as respiratory bronchiolitis-associated ILD (RB-ILD) and desquamative interstitial pneumonia (DIP) emphasize the impact of tobacco exposure on disease pathogenesis^3^.

Given the evidence linking smoking to ILDs, immediate efforts are needed to implement comprehensive strategies for tobacco cessation, public awareness, and early ILD detection among such high-risk populations. By further elucidating the mechanisms underlying the smoking-ILD association, future research endeavors hold promise for enhancing clinical outcomes and improving the quality of life for individuals afflicted by these debilitating lung conditions.

This review aims to discuss the main interstitial lung diseases linked to smoking, the impact cigarette use has on the development, progression, and prognosis of these entities, and also provide insight into tobacco cessation tailored to this special category.

## THE EFFECT OF SMOKING IN PATIENTS WITH ILDS

Tobacco smoke contains harmful particles and chemicals that trigger inflammation and damage lung cells, from airways to gas exchange regions, by inducing the release of inflammatory molecules and recruiting immune cells. Emphysema, a common smoking-related lung injury, involves cell death and loss of lung structure, sharing mechanisms with fibrosis. Alveolar epithelial stem cells, specifically type 2 alveolar epithelial cells, are essential in smoking-related lung diseases such as emphysema and fibrosis. Telomere dysfunction and cellular senescence contribute to susceptibility to these conditions. Cigarette smoke induces alveolar epithelial cell senescence, leading to a senescence-associated secretory phenotype that releases inflammatory mediators implicated in emphysema and fibrosis pathogenesis. Direct induction of profibrotic factors like transforming growth factor beta 1 (TGF-β1) and platelet-derived growth factor (PDGF) by cigarette smoke further promotes tissue remodeling. TGF-β1 stimulates matrix generation by fibroblasts, while PDGF enhances fibroblast proliferation^4^.

Cigarette smoking triggers various abnormalities in lung cells, implicating them in smoking-related ILD pathogenesis. Constituents in smoke activate epithelial cells, macrophages, neutrophils, and dendritic cells, prompting the release of inflammatory mediators. This cascade may be heightened in smokers with ILD, leading to sustained inflammation. The role of anti-inflammatory mechanisms and external factors like viral infections warrants further investigation^5^.

Moreover, cigarette smoke can induce acute lung injury, resulting in acute eosinophilic pneumonia, by recruiting and activating eosinophils. Eosinophilic granule constituents, such as cytokines and leukotrienes, lead to tissue damage and edema and stimulate mast cell and T lymphocyte activity^6^.

## SMOKING-RELATED INTERSTITIAL LUNG DISEASES

Although its harmful effects can be attributed to various diseases, cigarette smoking is a major risk factor and plays an important pathogenetic role in interstitial lung diseases from all points of view. Smoking-related interstitial lung diseases encompass acute eosinophilic pneumonia (AEP), respiratory bronchiolitis interstitial lung disease (RB-ILD), desquamative interstitial pneumonia (DIP), idiopathic pulmonary fibrosis (IPF), and combined pulmonary fibrosis emphysema (CPFE). All of these entities have different pathologic mechanisms, which results in various airway changes and different imagistic patterns^7^.

Cigarette smoking plays the principal etiologic factor in several ILDs, such as respiratory bronchiolitis-associated ILD, desquamative interstitial pneumonia (DIP), pulmonary Langerhans cell histiocytosis (PLCH), and it stands as a strongly recognized risk factor for idiopathic pulmonary fibrosis (IPF) and rheumatoid arthritis associated ILD^5^.

### Acute eosinophilic pneumonia

Acute eosinophilic pneumonia (AEP), a non-infectious pneumonia, is predominantly seen in smokers, with an increased risk for new-onset smokers, former smokers who have restarted smoking, and smokers who increase the number of cigarettes smoked daily. Moreover, recurrence often follows changes in smoking habits^8,9^.

Eosinophils, descending from the bone marrow, are vital for immune defense. Their intracytoplasmatic granules, with proteins, cytokines, and lipid mediators, exhibit an important role in defending against pathogens. However, excessive activation can lead to tissue damage. Acute eosinophilic pneumonia (AEP) exemplifies this, where increased eosinophil proliferation and activity result in extensive tissue injury, highlighting the balance of the immune response^8,9^.

Suspected pathogenesis also involves IL-5-mediated inflammation due to inhaled cigarette toxins. The eosinophils’ impact on basal membranes causes fluid and protein leakage into air spaces, resulting in diffuse alveolar damage. Imaging shows nonspecific findings like consolidation, ground glass opacity, nodules, and pleural effusions. Distribution is typically peripheral or random. Smoking cessation can trigger eosinophilia recurrence in bronchoalveolar lavage (BAL) samples. BAL also shows elevated eosinophils and cytokine levels, which correlate with smoking exposure^7,10,11^.

### Respiratory bronchiolitis - ILD

Inhalation of cigarette smoke triggers various immune responses in the lungs, one of which is respiratory bronchiolitis (RB), characterized by increased production of macrophages in the distal bronchioles and alveoli. These macrophages, also called „smokers’ macrophages,” exhibit a phagocytic capacity by inhaling particles, resulting in fine granular pigment accumulation. Despite being found in approximately all smokers, it may not always manifest on CT scans, even if, from a histological point of view, it is described in nearly all smokers^12,13^.

Respiratory bronchiolitis is usually asymptomatic, but its severe form presents with dyspnea, cough, and wheezing. Diagnosis relies on smoking history, symptoms, pulmonary function testing, and imaging. The HRCT examination identifies centrilobular nodules, which frequently appear blurry, sharing imaging characteristics similar to the inflammatory subacute stage of hypersensitivity pneumonitis. Surgical or transbronchial biopsies are rarely needed. Smoking cessation is a key intervention, often leading to clinical and radiographic improvement without further intervention^7^.

So, what is the difference between respiratory bronchiolitis and respiratory bronchiolitis-interstitial lung disease (RB-ILD)? RB-interstitial lung disease (RB-ILD) refers to RB with clinical symptoms like cough and shortness of breath, often overlapping with desquamative interstitial pneumonia (DIP). RB-interstitial lung disease (RB-ILD) is diagnosed when respiratory bronchiolitis histologic and imaging findings align with clinical symptoms and pulmonary function abnormalities^14^.

### Desquamative interstitial Pneumonia

The histological condition called desquamative interstitial pneumonia (DIP) is frequently used when alveoli are extensively filled with „smokers’ macrophages.” The differentiation between RB and DIP can be quite challenging and unclear. DIP is rare, affecting individuals aged 40-60, with a male predominance. Smoking is prevalent in almost all cases. Histologically characterized by smoker macrophages, DIP manifests with exertional dyspnea and cough. HRCT examination shows ground-glass opacities, predominantly in the lower lobes, separated by normal lung, which creates the radiological aspect of mosaic attenuation, along with emphysema. The diagnosis, often requiring a biopsy, may be challenging. Smoking cessation is crucial, with corticosteroids as standard therapy. For those in whom lung fibrosis is present, standard therapy may not be sufficient, and the fibrotic disease can progress, leading to respiratory failure^7,8,15,16^.

3.4 Pulmonary Langerhans Cell Histiocytosis Pulmonary Langerhans Cell Histiocytosis (PLCH) also represents a disease mainly related to smoking, which affects young adults and is estimated to include 3-5% of diffuse parenchymal lung diseases. Langerhans cells, a subset of dendritic cells, are only found in a few organs, such as the skin, thymus, and airways, serving as the primary defense against inhaled antigens in the respiratory system. Even if Langerhans cells represent a normal finding of the cells residing in the airways, their numbers in the smoker population are elevated, and interestingly enough, only a small percentage develop the disease^17,18^.

Cigarette smoke triggers dendritic cell accumulation, leading to characteristic histopathological findings.

Another proposed mechanism is the genetic theory, taking into account the fact that not all smokers develop PLCH. Symptoms include cough, dyspnea, and extrapulmonary manifestations. HRCT examination reveals stellate nodules in upper and mid-lung zones, with a diameter of less than 1 cm, with or without the presence of cavitary nodules. Over time, they can merge into larger cysts surrounded by fibrous tissue, resembling emphysema. Bronchoscopy confirms the diagnosis. Smoking cessation represents the primary treatment and is beneficial for 30% of patients while helping symptomatic cases^7,19,20,21^.

### Idiopathic Pulmonary Fibrosis

When discussing interstitial lung diseases, maybe one of the most severe and with the poorest prognosis remains idiopathic pulmonary fibrosis (IPF). Cigarette use can represent a risk factor for the development of IPF, and also smoking represents a major risk factor in the evolution of IPF, has the potential to modify the course of the disease, and has been associated with a poorer prognosis. Moreover, individuals who smoke tend to develop the disease earlier compared to non-smokers, with a median age of onset at around 60-70 years, and they also survive less in comparison to non-smokers^22,23,24,25^.

With that being said, the pathogenic significance and mechanisms are still quite uncertain. A study by Antoniou in 2008 evaluated patients with idiopathic pulmonary fibrosis in relation to their smoking status regarding survival. The results were surprising – among individuals with IPF, nonsmokers exhibited higher survival rates and severity-adjusted survival compared to former smokers. However, apparently, better outcomes were observed in current smokers relative to former smokers, a result which likely represents a less extensive disease at presentation and may reflect a phenomenon known as the „healthy smoker effect”^26^.

The „healthy smoker” effect refers to the phenomenon wherein current smokers may appear to have better health outcomes compared to former smokers or non-smokers despite the known detrimental effects of smoking on overall health. The effect of being a healthy smoker results from various factors, such as socioeconomic factors, access to healthcare, and healthy behaviors, which have a great impact on perceived health. Moreover, some smokers may possess genetic or lifestyle advantages, delaying health deterioration. Despite these complexities, long-term smoking risks remain significant, emphasizing the importance of cessation in preventive healthcare^27^.

Another study by Antoniou in 2013 brought to light again the link between smoking and ILDs. This time, the aim of the study was to determine the presence of emphysema and to quantify its extension in patients with idiopathic pulmonary fibrosis and rheumatoid arthritis-interstitial lung disease (RA-ILD). The study revealed a surprisingly high prevalence of emphysema among smokers with IPF and RA-ILD despite their relatively low pack-year smoking histories. This finding highlights potential shared pathogenetic pathways between smoking-related lung damage and fibrotic lung diseases. Additionally, smokers with IPF exhibited more prominent honeycombing and coarse reticulation, suggesting a potential association between smoking and disease severity^28^.

### Combined Pulmonary Fibrosis and Emphysema

This study also features the additional injuries cigarette smoking can cause in an already injured lung. However, the association of lung emphysema and pulmonary fibrosis was highlighted in a review by Oliva et al. in 2011 and by Cottin et al. in 2005 and later in 2023, who suggested an acronym for this association between the two – CPFE – combined pulmonary fibrosis and emphysema. Initially, the coexistence of pulmonary fibrosis and emphysema was considered incidental, with unclear links between their pathogenesis. Animal studies suggested cigarette smoke could trigger both conditions, but this hasn’t been proven in humans. However, research indicates a higher prevalence of pulmonary fibrosis in smokers, hinting at shared factors^29^.

In a study by Cottin et al. in 2005, 61 CPFE patients were examined, predominantly male smokers, with a median diagnosis age of 65 and with key features mirroring IPF. CT scans revealed interstitial pneumonia, often challenging IPF diagnosis. Lung function tests showed near-normal volumes but a severely impaired CO transfer. Median survival was 6.1 years, worse than emphysema alone but better than IPF. Nearly half had pulmonary hypertension, a significant predictor of survival. Smoking likely plays a central role in CPFE pathophysiology, and treatments show limited efficacy, highlighting the importance of smoking cessation. CPFE presents a distinct entity with unique functional profiles and prognosis, requiring careful diagnosis and management^30^.

The topic was later reevaluated by the same author in 2023, with a comprehensive review of CPFE in which the following key points were highlighted - CPFE predominantly affects male smokers, with an average exposure of 40 pack-years. Smoking history is more common in CPFE compared to isolated IPF or systemic sclerosis-associated ILD. Patients with CPFE exhibit limited exercise capacity, severely impaired diffusing capacity of the lungs for carbon monoxide (DLco), and carbon monoxide transfer coefficient (Kco), contrasting with preserved airflow rates and lung volumes. Compared to isolated IPF, CPFE patients have higher lung volumes, lower DLco, and lower PaO2. Relative preservation of flow rates and lung volumes is attributed to the counterbalancing effects of restrictive physiology from pulmonary fibrosis and emphysema. CPFE patients experience slower FVC decline, but no reliable parameter exists to monitor disease progression. Monitoring CPFE progression should involve clinical imaging and multiple functional parameters. One of the complications reported in the review is pulmonary hypertension, with a prevalence in CPFE that ranged from 15-55%. Pathophysiology is likely multifactorial, with worse severity compared to IPF or COPD. Another complication was represented by lung cancer, with an estimated risk of 2-52%, with a higher prevalence than IPF alone. Squamous cell carcinoma and adenocarcinoma were common in these patients, and it is also associated with worse lung cancer survival^31,32,33^.

A study by Mitchell et al. in 2014 aimed to examine smoker patients with IPF and other forms of usual interstitial pneumonia (UIP) from a radiological point of view and to compare radiologic differences in the severity of fibrosis and emphysema. IPF smokers exhibited greater lung fibrosis and reticulation scores compared to non-IPF UIP smokers. Smokers with IPF and emphysema showed worse radiologic combined pulmonary fibrosis emphysema (CPFE) findings than those with non-IPF UIP/emphysema^34^.

### Smoking-related Related Interstitial Fibrosis

Another pathologic entity in relation to smoking was documented and posed a high interest – smoking-related interstitial fibrosis (SRIF). SRIF is a term that describes a specific pattern of interstitial fibrosis often observed alongside emphysema in individuals who smoke. This topic was intensely researched by Katzenstein et al. in 3 different articles in 2010, 2012, and 2013. Distinguishing SRIF from usual interstitial pneumonia (UIP), fibrosing non-specific interstitial pneumonia (NSIP), and other entities relies on specific histological features. Unlike UIP, SRIF lacks honeycomb changes, exhibits characteristic hyalinized fibrosis, and is consistently associated with emphysema and RB. Differential diagnosis with fibrosing NSIP considers the nature of fibrosis, degree of inflammation, and distribution of changes. RBILD/DIP spectrum, though sharing similarities, differs from SRIF by its minimal interstitial fibrosis. Similarly, scarred Langerhans Cell Histiocytosis (LCH) may overlap with SRIF but presents distinctive peribronchiolar scarring. Combined emphysema and pulmonary fibrosis cases often pose diagnostic challenges but may include SRIF^35,36,37^.

Clinically, SRIF is often incidental, detected during lung tissue examination for other conditions, with mild obstructive defects being the most common functional abnormalities. While rarely symptomatic, some patients present with respiratory complaints, though their prognosis remains favorable without evidence of progressive impairment. HRCT examination reveals the presence of reticulation, with or without ground-glass lesions predominantly in the upper areas of the lung and lesions of emphysema^35,36,37,38,39^.

Although it can seem overwhelming from a clinical point of view and can pose a significant challenge even for experienced ILD specialists, it’s crucial to approach each case with a multidisciplinary team, including pulmonologists, radiologists, pathologists, and rheumatologists, to ensure accurate diagnosis and optimal management tailored to the individual patient’s needs.

## SMOKING CESSATION – WHY, WHEN AND HOW?

As illustrated above, smoking cessation plays a major role in the prognosis of smoking-associated ILDs. Smoking cessation stands as a crucial therapeutic intervention for individuals with smoking-related ILDs, presenting a key opportunity to mitigate disease progression and improve clinical outcomes. Physicians play a vital role in advocating for smoking cessation among all patients who smoke, as evidence suggests that physician advice significantly enhances the likelihood of successful quitting. Despite the proven effectiveness of smoking cessation programs in facilitating abstinence, the addictive nature of nicotine poses quite a challenge, even for motivated individuals. Tobacco dependence often manifests as a chronic, relapsing condition, with the majority of smokers crossing prolonged periods of tobacco use combined with several attempts to quit. Despite these obstacles, sustained efforts to support smoking cessation remain crucial in the management of smoking-related ILDs, offering evidence-based benefits to patients’ good health status and well-being^40^.

To support these statements, Choi et al. conducted 2018 a longitudinal population study spanning nine years, evaluating risk factors contributing to ILD development among people aged 40 and above. Out of 312,519 individuals included, 1972 developed ILD during the study period, yielding an incidence rate of 70.1 cases per 100,000 individuals annually. Moreover, a multivariate analysis revealed a significant association between smoking and ILD onset^41^.

Research on smoking cessation in interstitial lung diseases (ILDs) is sparse despite their significant impact on lung health. The limited available studies hinder the understanding of tailored cessation approaches for ILD patients, delaying effective interventions. Addressing this issue is essential to improve ILD management, emphasizing the need for further research (Table 1).

**Table 1:** A summary of main interstitial diseases associated with cigarette smoking^4,5^

*Characteristic*	*RB-ILD*	*IPF*	*DIP*	*SRIF*	*PLCH*	*AEP*	*CFPE*
Symptoms	Cough Dyspnea	Dry cough Progressive dyspnea	Cough, dyspnea, cough, fatigue	Cough, dyspnea	Cough,dyspnea, extrapulmonary manifestations	Acute onset with respiratory distress Cough, dyspnea	Cough, Dyspnea
Radiological findings	Centrilobular nodules Ground-glass opacities Distribution usually in the upper lobes	Honeycombing pattern Reticulation Traction bronchiectasis Distribution predominantly in the lower lobes	Ground glass opacities, nodules	Centrilobular nodules Ground-glass opacities	Nodules and cysts Costophrenic angle is spared Distribution predominantly in the upper lung zones	Diffuse bilateral ground-glass opacities and reticulation	Emphysema predominantly in the upper lobes Fibrosis predominantly in the lower lobes
Treatment options	***Smoking cessation*** Supportive care Corticotherapy in refractory disease	***Smoking cessation*** Antifibrotic therapy Lung transplant	***Smoking cessation*** Supportive care Corticotherapy in refractory disease	***Smoking cessation*** Supportive care Corticotherapy in severe disease	***Smoking cessation*** Targeted therapy Lung transplant for severe disease	***Smoking cessation*** Corticotherapy	***Smoking cessation*** Lung transplant

The impact of smoking cessation programs on interstitial lung disease (ILD) prognosis remains unclear, with no specific treatment recommendations in ILD guidelines. A retrospective analysis by Garcia et al. included 142 smokers who were treated at a high-complexity tobacco addiction unit between June 2018 and February 2021. The study revealed that 15 (10.56%) had interstitial lung disease (ILD). Of the 15 included patients, 53% were female, with a mean age of 61 years. They began smoking after 19 years old, with a mean daily consumption of 20 cigarettes and an average cumulative pack-year dose of 34. On average, they attempted to quit smoking 1.6 times, achieving a maximum abstinence period of 14 months. Additionally, 87% had comorbidities. Questionnaire results showed moderate nicotine dependence.). Varenicline was the most common therapy used for smoking cessation in 53% of the patients, followed by nicotine replacement therapy in 40% of the cases. 13% received no treatment, and 7% used a combination of varenicline and NRT. Exhaled carbon monoxide (CO) levels were measured at the initial visit, three months, and 6 months post-quit day. Abstinence rates were 53% at three months and 27% at six months. The most frequent relapse triggers were stress in 47% and social interactions with smokers in 20% of the cases. Follow-up attendance rate at six months was 87%^42^.

In a retrospective cohort study bu Ash et al. 8266 participants from the COPDGene study (October 2006 - January 2011) were analyzed using CT to measure interstitial features and emphysema. Results showed that smokers with both emphysema and interstitial features had worse outcomes lower DLCO, shorter 6-minute walk distance, higher SGRQ scores, and increased mortality. Interstitial features significantly worsened the clinical severity and mortality associated with emphysema^43^.

**Figure 1:**
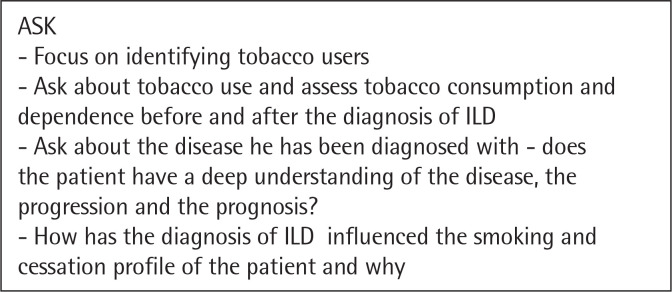
The Five A’s – ASK^44^

**Figure 2:**
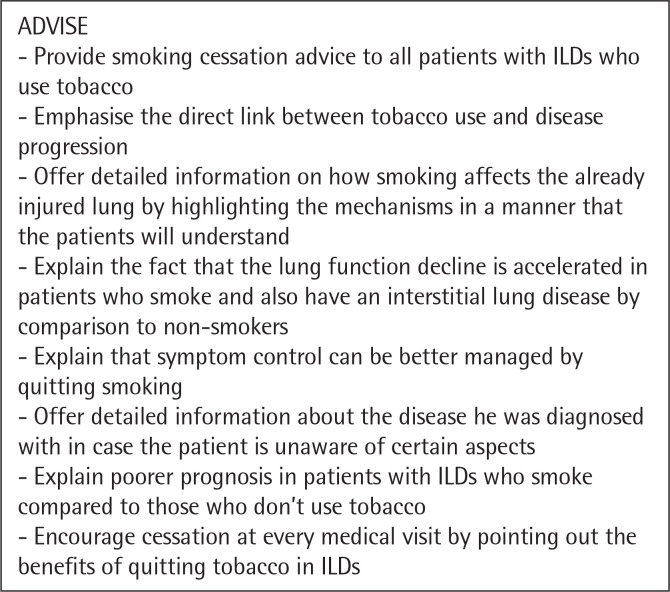
The Five A’s - ADVISE^44^

**Figure 3:**
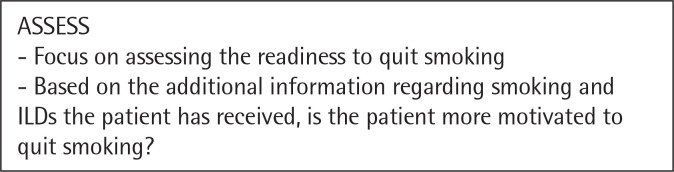
The Five A’s – ASSESS^44^

**Figure 4:**
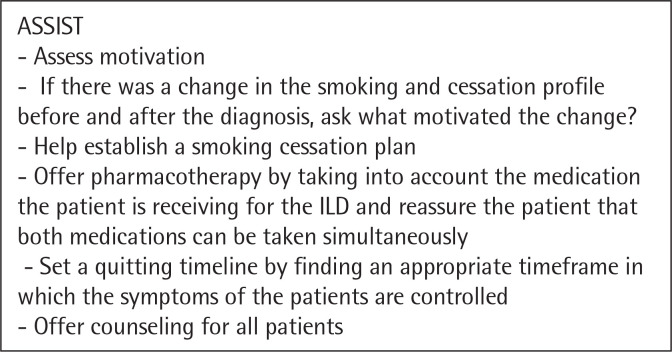
The Five A’s - ASSIST^44^

**Figure 5:**
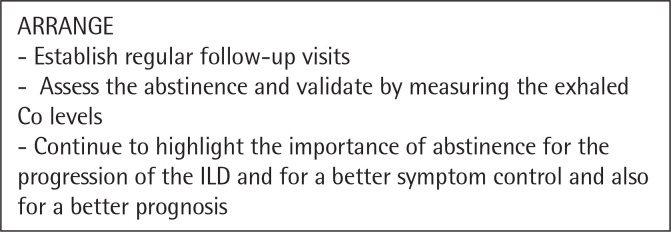
The Five A’s – ARRANGE^44^

The need for a tailored approach and intervention for smoking cessation in patients with interstitial lung disease is paramount. Clinicians should still follow the „5 A” strategy, but it should be managed accordingly to the disease pattern^44^. We propose a personalized approach for patients with ILDs illustrated in the following figures (Figures 1, 2, 3, 4, 5). However there are not many studies showcasing a personalised approach to quitting smoking in patients with ILDs. Thid fields requires further studies in order to better understand what could motivate a patient with ILD to quit smoking and maintain abstinence.

All patients with ILD should be routinely inquired about their smoking habits. This guidance should be explicitly communicated and tailored to each individual. Clinicians who treat smokers diagnosed with ILDs are managing a condition which could have been a result of cigarette use and moreover worsened by smoking. It is important for both the clinician and the patient to highlight the fact that their disease is linked to smoking and quitting will have a beneficial health effect and could contribute to a slower progression of the disease^40^.

A study by Sawata et al. in 2016 which included 31 patient diagnosed with NSIP analyzed pulmonary function tests, other biological markers and clinical course over two years by dividing them into 2 groups – smokers and non-smokers. In the smoker group patients had lower diffusing capacity for carbon monoxide/alveolar ventilation (DLCO/VA) values compared to the non-smoker group. This study shows a significant negative impact in patients with NSIP pattern, exacerbated by smoking. However the study is limited and the prognosis could not be appreciated^45^. It is also noteworthy to advise the patient on other health risks tobacco use has. Putman et al. conducted a lung cancer screening trial in 2016 on 4101 individuals between the ages of 50-70 with a history of smoking of at least 20 PACK-YEARS. From this group, 1,920 patients were included in a subsequent analysis to examine whether incidental interstitial lung abnormalities observed in this cohort were associated with increased mortality over a 12-year follow-up. Among these individuals, 16.7% had interstitial involvement and they also had a higher all-cause mortality compared to those without interstitial involvement, regardless of their ILD histologic pattern. The results showed an increased risk of death from lung cancer and non-pulmonary malignancies. Considering this, the clinician should not focus only on the progression of the ILD but also on the other organs and systems that tobacco can negatively affect^46^.

The clinician should take into account the type of ILD’s the patient has been diagnosed with. For example an acute manifestation of the disease such as acute eosinophilic pneumonia with symptoms that quickly appeared, may make the patient more inclined to quit smoking in order to return to a former health status. However, patients with chronic symptoms, for example patients with IPF or CPFE in whom the symptoms developed over several months or years, may make smoking cessation much more difficult. Clinicians should also offer examples of changes that could happen if the patient quits smoking. Nakanishi et al. in a retrospective study analyzed five patients with respiratory bronchiolitis-associated interstitial lung disease,who were diagnosed via surgical biopsy. The only treatment was smoking cessation; no steroids or immunosuppressive therapies were used. All the patients had smoking histories. After the diagnosis, all patients quit smoking, and they were followed for a period of time of 5 to 62 months. All patients showed symptom improvement after toacco cessation, the CO diffusing capacity and oxigenation improved. Also all patients showed radiological improvement^47^.

Even with a well-appointed plan, the patient could relapse. It is important for the clinician to closely monitor the patient and identify the triggers that could cause a relapse such as Life stressors, anxiety, emotional difficulties regarding their disease, lack of support from the family or even depression or mental health issues.

## CONCLUSIONS

Smoking cessation is especially important for individuals suffering interstitial lung diseases (ILDs) due to the exacerbating effects of tobacco use on these conditions. Continued smoking worsens ILDs, accelerating disease progression and increasing the risk of complications. Addressing the challenges of smoking cessation outlined above is crucial in this context, as quitting smoking can significantly slow the progression of ILDs and improve overall lung function. Therefore, personalized strategies in order to target both nicotine dependence and psychosocial factors are essential for patients with ILDs to achieve successful smoking cessation and to reduce the impact of smoking on their lung health.

## Data Availability

The data supporting this research are available from the authors on reasonable request.
